# Periodontal therapy mitigates inflammation via TNF-α reduction and improves glycolipid metabolism in diabetic patients: a cross-sectional cohort study

**DOI:** 10.3389/fendo.2025.1668695

**Published:** 2025-10-30

**Authors:** Yiman Guo, Xincheng Zhang, Aige Yang, Yuqing Guo, Shanshan Dong, Lina Wang, Ning Zhang, Huimin Zhou

**Affiliations:** ^1^ Department of Orthodontics, Beijing Stomatological Hospital and School of Stomatology,Capital Medical University, Beijing, China; ^2^ Department of Endocrinology, First Hospital of Hebei Medical University, Shijiazhuang, China; ^3^ Department of Endocrinology, Third Hospital of Hebei Medical University, Shijiazhuang, China; ^4^ Clinical Research Center for Endocrine and Metabolic Diseases, Shijiazhuang, China

**Keywords:** diabetes mellitus, periodontitis, tumor necrosis factor α, glycosylated hemoglobin, resistin

## Abstract

**Objective:**

To evaluate the effects of non-surgical periodontal therapy on systemic and local levels of tumor necrosis factor-alpha (TNF-α) and their relationship with glycolipid metabolism in patients with type 2 diabetes mellitus (T2DM) and periodontitis.

**Methods:**

In this prospective cohort study, 234 patients with T2DM and periodontitis underwent standardized non-surgical periodontal therapy, including scaling and root planing, supplemented with systemic antibiotics. Clinical parameters (gingival bleeding index, periodontitis staging and grading), serum levels of TNF-α, HbA1c, leptin, adiponectin, resistin, free fatty acids (FFA), and levels of TNF-α, leptin, adiponectin, and resistin in gingival crevicular fluid (GCF) were assessed at baseline, 4 weeks, and 8 weeks post-treatment. Correlations were analyzed using Spearman’s rank correlation with false discovery rate correction.

**Results:**

Periodontal therapy resulted in significant improvements in all clinical periodontal parameters (P<0.01). Systemic and local GCF levels of TNF-α, leptin, resistin, and FFA demonstrated significant and progressive reductions, while adiponectin levels increased significantly at 4 and 8 weeks (P<0.01). HbA1c and FBG levels were also significantly improved by week 8 (P<0.01). TNF-α dynamics were strongly correlated with adipokine levels and clinical indices, with the most robust correlations observed within the GCF microenvironment.

**Conclusion:**

Systematic periodontal therapy effectively reduces local and systemic inflammation, improves glycemic control, and ameliorates glycolipid metabolic disorders in patients with T2DM and periodontitis. The strong interrelationships observed, particularly within the GCF, underscore the potential role of TNF-α as a key mediator in the mouth-systemic metabolic interplay.

## Introduction

Diabetes mellitus and periodontitis are both highly prevalent chronic diseases with a strong bidirectional relationship (1). The long-term hyperglycemic state in diabetic patients reduces their resistance to infection and ability to heal, making them prone to various complications—with periodontitis being one of the most common oral manifestations (2). Research indicates that periodontitis occurs more frequently in diabetic patients than in non-diabetic populations, with its severity directly correlating to glycemic control levels (3). As a chronic infectious condition, periodontitis damages periodontal tissues (gingiva, periodontal ligament, dentin and alveolar bone). Its pathogenic bacteria and inflammatory mediators can spread throughout the body via the bloodstream, elevating the risk of diabetes mellitus and cardiovascular disease (4–6). Furthermore, periodontitis impacts glycemic control in diabetic patients and may increase their risk of other complications (7). Understanding the mechanisms of interaction between diabetes and periodontitis is therefore crucial for early intervention and comprehensive patient management.

In recent years, periodontitis has been considered a localized inflammatory disease of the oral periodontal tissues (8). However, growing research shows that diabetes and periodontitis share a common pathogenic mechanism—both involve pro-inflammatory mediators in the inflammatory response (9, 10). Periodontitis has strong associations with several systemic diseases, including cardiovascular disease, blood disorders, chronic liver disease, osteoporosis, and autoimmune diseases (11). It has also emerged as the sixth most common complication of diabetes mellitus (12). The oral microbiome plays a crucial role in how both periodontitis and diabetes mellitus develop and progress by modulating the innate and adaptive immune systems (13, 14). Additionally, the buildup of cytokines, chemokines, prostaglandins, and late glycosylation end products is central to the disease’s pathogenesis and progression (15, 16). Treatment of periodontitis has shown notable benefits—it significantly improves glycated hemoglobin (HbA1c) levels in diabetes patients with poor glycemic control (17). These findings indicate that the interaction between periodontitis and diabetes mellitus extends beyond oral health, significantly affecting systemic metabolic status. This highlights the critical role of oral health in managing chronic diseases.

Tumor necrosis factor α (TNF-α), a major inflammatory factor, plays a dual role in both the inflammatory response of periodontitis and glycolipid metabolism (18). Studies have shown that TNF-α induces insulin resistance and disrupts lipid metabolism, leading to glucose and lipid abnormalities (19). However, TNF-α’s mechanism of action in patients with diabetes mellitus and periodontitis remains unclear, particularly regarding its relationship with glycolipid metabolism indices before and after periodontal treatment. Prolonged TNF-α expression may represent a pathway through which bacteria cause significant inflammatory damage, a process sometimes enhanced by immunodeficiency from lymphocyte subset mobilization (20). Hyperglycemia can worsen periodontal disease in diabetic patients by affecting TNF-α levels (21). The systemic nature of this inflammatory response is evidenced by elevated serum TNF-α levels in patients with acute apical abscesses (22). Additionally, adipokines—including lipocalin and resistin—secreted by adipocytes have emerged as prognostic markers of oral disease (23). In this study, we hypothesized that periodontal treatment could significantly reduce TNF-α levels and thereby improve glycolipid metabolism. By analyzing the correlation between TNF-α and glycolipid metabolism indicators, this study aims to provide a scientific basis for clinical treatment and further clarify the interaction between diabetes and periodontitis.

## Information and methods

### Case selection

This cross-sectional cohort study aimed to examine the correlation between TNF-α and glycolipid metabolic indices in patients aged 18–75 years with diabetes mellitus complicated by periodontitis. Data of 234 patients with diabetes mellitus and periodontitis were collected from March 2022 to April 2024 in our hospital. Patients underwent basic therapies, including scaling, root planing, and conventional medication. The study followed the Declaration of Helsinki guidelines and was approved by our hospital’s medical ethics committee.

Inclusion criteria: (1) Diagnosed with type II diabetes mellitus (T2DM) according to the American Diabetes Association (ADA) 2022 criteria (24). Patients were required to have a documented disease duration of ≥1 year and stable glycemic control, defined as no change in hypoglycemic medication regimen for at least 3 months prior to enrollment. Based on baseline HbA1c levels, diabetic control was categorized as: inadequate control (HbA1c 7.5%–9.0%) and moderate control (HbA1c <7.5%) (25). The majority of the cohort fell into the inadequate control category to investigate the metabolic effects of periodontal therapy in this challenging population. (2) ≥15 natural teeth present (excluding third molars and retained roots). (3) & (4) A diagnosis of periodontitis was established according to the 2018 World Workshop on the Classification of Periodontal and Peri-Implant Diseases and Conditions (26). Periodontitis Staging: All enrolled patients were classified as either Stage III (severe periodontitis with potential for tooth loss due to periodontitis) or Stage IV (advanced periodontitis with extensive tooth loss and complex rehabilitation needs), based on interdental CAL (≥5 mm at the maximum site), radiographic bone loss extending to the mid-third of the root or beyond, and probing depth (PD) ≥6 mm at the maximum site. Periodontitis Grading: The grade of periodontitis was determined primarily based on the evidence of progression, calculated as the percentage of bone loss divided by the patient’s age (BL/A) (26). Since all subjects were diagnosed with diabetes mellitus—a well-established risk factor for periodontitis progression—the grade was directly modified to Grade B (moderate progression rate) or Grade C (rapid progression rate), as per the classification guidelines. (5) Age 18–75 years; (6) complete clinical data.

Exclusion criteria were rigorously applied to minimize confounding variables and ensure the internal validity of the study. These criteria were as follows: (1) Receipt of any periodontal therapy, including scaling and root planing, surgical procedures, or antibiotic treatment specifically for periodontal conditions, within the 6 months preceding the study baseline (27); (2) Chronic use (defined as >3 months) of medications known to affect periodontal tissues, such as phenytoin, calcium channel blockers, or cyclosporine, which can induce gingival enlargement (28); (3) Use of immunosuppressants or systemic corticosteroids within the 3 months prior to enrollment; (4) Presence of severe diabetic complications, including advanced retinopathy (confirmed by fundoscopy or OCT), nephropathy (defined as an eGFR <60 mL/min/1.73 m² or albuminuria >30 mg/g creatinine), or established neuropathy (Toronto Clinical Scoring System score ≥5), as these conditions can independently modulate systemic inflammation (29); (5) Concurrent active infections (e.g., pneumonia, urinary tract infection) or any malignancy; (6) Diagnosis of other systemic inflammatory or autoimmune diseases, such as rheumatoid arthritis or Crohn’s disease; (7) Poorly controlled hypertension (resting blood pressure ≥160/100 mmHg) or hyperlipidemia (LDL cholesterol ≥190 mg/dL) (29); (8) Significant smoking history, defined as ≥10 pack-years or current smoking status confirmed by a positive cotinine test, as smoking is a primary risk factor for periodontal disease progression (26); (9) Unstable glycemic control that necessitated frequent adjustments to medication.

The sample size was calculated using the standard formula for cross-sectional studies (30). As in Equation 1:


(1) $$n=\frac{Z_{1-\alpha /2}^{2}\times \left[p\left(1-p\right)\right]}{\delta^{2}}$$

Where n is the sample size, take α=0.05, check the Z-value table to get Z1-α/2 = 1.96, δ is the allowable error, control the allowable error at 6%, p denotes the prevalence of diabetes mellitus with periodontitis, according to the reference, the prevalence of diabetes mellitus with periodontitis is 73.14%, so the minimum total sample content is 218, and the sample size of the present study is 234 in accordance with the requirements.

### Intervening method

The periodontal treatment cycle lasted 4 weeks and included full-mouth cleaning, subgingival scaling, root planing, occlusal adjustment, and extraction of non-retainable affected teeth. During treatment, patients received oral tetracycline (1 g/d) for 2 weeks. The systematic basic periodontal treatment was delivered in 4 sessions at 1-week intervals, comprising supragingival scaling, subgingival scaling, and root planing. Patients received instruction in oral hygiene practices, including proper brushing techniques, flossing, and interproximal brushing. Throughout the study, patients maintained their usual diabetes medications. Along with dietary control and exercise, patients continued their prescribed medications: metformin (25 mg after meals, thrice daily or 50 mg, twice daily), glibenclamide (2.5 mg before meals, thrice daily), or thirst-quenching pills (10 capsules before meals, thrice daily), based on their individual conditions.

### Data collection

#### Tests for serological indices

Blood samples were collected at baseline (before treatment) and at 4 and 8 weeks post-treatment to assess systemic changes. All procedures were standardized to minimize pre-analytical variability, which is critical for the reliability of biomarker measurements (31). (1) Serum Sampling and Processing: A 2 mL sample of peripheral venous blood was drawn into an Eppendorf tube. The sample was allowed to clot at room temperature for 30 minutes before being centrifuged at 1,500 x g for 15 minutes at 4°C. The resulting serum was aliquoted and stored at -20°C until analysis. (2) Serum Metabolic and Adipokine Assays: A separate 10 mL sample of fasting venous blood was collected for metabolic panels. Fasting blood glucose (FBG) was measured using the glucose oxidase method, and glycated hemoglobin (HbA1c) was measured via immunosuppressive turbidimetry (Roche Diagnostics). Serum concentrations of leptin were determined by an immunoscattering turbidimetric assay (Shanghai Debo Biotechnology Co.). Adiponectin and resistin levels were quantified using commercially available enzyme-linked immunosorbent assay (ELISA) kits (Shenzhen Jingmei Biological Co., Ltd. and Shenzhen Xinbosheng Biotechnology Co., respectively). All assays were performed according to the manufacturers’ detailed instructions. (3) Serum Free Fatty Acids (FFA): FFA levels were measured using a photometric colorimetric method on a fully automated spectrophotometer (TAS-990, Beijing Purkinje General Instrument Co., Ltd.).

#### Gingival crevicular fluid sampling and analysis

To assess the local periodontal inflammatory and metabolic milieu, gingival crevicular fluid (GCF) was collected at baseline, 4 weeks, and 8 weeks post-treatment, coinciding with the serum sampling time points. The sampling protocol was adapted from established methods (32). Briefly, GCF was obtained from the four sites with the deepest probing depths (one site per quadrant) using standardized periopaper strips (OraFlow Inc., USA). Sites were isolated with cotton rolls, supragingival plaque was carefully removed, and the area was gently air-dried. Paper strips were inserted into the gingival sulcus until mild resistance was felt and left in place for 30 seconds. Strips contaminated with blood or saliva were discarded. The GCF volume was immediately measured using a pre-calibrated electronic periotrometer (Periotron 8000, OraFlow Inc., USA). Each strip was then eluted into 200 μL of phosphate-buffered saline and stored at -80 °C until batch analysis. Levels of TNF-α, leptin, adiponectin, and resistin in the GCF eluent were determined using commercially available, high-sensitivity ELISA kits (R&D Systems, USA), following the manufacturers’ instructions. The total amount of each analyte (pg) was normalized to the GCF volume (μL) and expressed as concentration (pg/μL) for statistical analysis.

#### Gingival bleeding index

The gingival bleeding index was assessed to quantify gingival inflammation (33). All clinical examiners underwent standardized training and calibration exercises before the study commenced to ensure high inter-rater reliability. Agreement was assessed using the Cohen’s kappa statistic, with a target value of κ > 0.8 indicating almost perfect agreement (34).

(1) Examination Procedure: A blunt-ended periodontal probe with a tip diameter of 0.5 mm was gently guided into the gingival sulcus, parallel to the long axis of the tooth. The probe was then swept from the distal to the mesial aspect along the sulcus base on both the buccal/labial and lingual/palatal surfaces of each tooth. After probing each tooth, the examiner waited for 10–15 seconds to observe any bleeding.

(2) Scoring Criteria: The following 0–5 scale was used to score bleeding for each tooth surface: 0 points: Healthy gingival margin and papilla; no bleeding after probing. 1 point: Apparently healthy gingiva, but bleeding appears from the sulcus upon probing. 2 points: Bleeding on probing with an accompanying change in gingival color. No swelling is observed. 3 points: Bleeding on probing, color change, and mild gingival edema. 4 points: Bleeding on probing, obvious color change, and significant edema. 5 points: Spontaneous bleeding or severe bleeding upon probing, accompanied by significant edema and color change, potentially with ulceration.

#### Periodontitis staging and grading

As detailed in the inclusion criteria, all patients were diagnosed, staged, and graded according to the 2018 International Classification of Periodontal Diseases (26). Staging (I-IV) defined the severity and complexity of the disease, while grading (A-C) estimated the rate of progression. The presence of diabetes mellitus was used as a modifier to upgrade the grade, reflecting the patient’s heightened risk for disease progression. All staging and grading assessments were conducted by calibrated periodontists based on full-mouth clinical and radiographic examinations.

Examiners were similarly trained and calibrated for periodontitis grading based on the 2018 International Classification of Periodontal Diseases. Inter-rater reliability was assessed with kappa statistics, showing good agreement (kappa > 0.8).

### Statistical methods

Statistical analyses were performed using IBM SPSS Statistics 25.0. The normality of continuous variables was assessed via the Shapiro–Wilk test (alpha=0.05), and homogeneity of variances was evaluated using Levene’s test. For longitudinal comparisons of normally distributed data that met the sphericity assumption (Mauchly’s test, p > 0.05), repeated-measures analysis of variance (ANOVA) was applied, followed by Bonferroni-adjusted pairwise comparisons for *post hoc* testing. When the sphericity assumption was violated (p ≤ 0.05), the Greenhouse–Geisser correction was used. For non-normally distributed longitudinal data, the Friedman test was employed, with Dunn–Bonferroni tests for subsequent pairwise comparisons when overall significance was detected. Normally distributed data are presented as the mean ± standard deviation (SD), and non-normally distributed data as the median (interquartile range, IQR). Categorical variables were analyzed using the chi-square test or Fisher’s exact test, as appropriate. Bivariate correlations were examined using Spearman’s rank correlation coefficient (ρ), with the Benjamini–Hochberg procedure applied to control the false discovery rate (FDR).

## Results

### Basic data of the patients

The baseline characteristics of the 234 enrolled patients are summarized in **Table 1**. The cohort comprised 130 males (55.6%) and 104 females (44.4%), with a mean age of 68.4 ± 6.1 years. According to the predefined glycemic control categories, the majority of patients (n=178, 76.1%) were classified as having inadequate diabetic control (HbA1c 7.5%–9.0%), while 56 patients (23.9%) were in the moderate control group (HbA1c <7.5%). Regarding periodontal status, all patients were diagnosed with severe or advanced periodontitis as per the 2018 classification, with 151 patients (64.5%) classified as Stage III and 83 patients (35.5%) as Stage IV. In terms of disease progression rate (Grading), 162 patients (69.2%) were graded as Grade C (rapid progression) and 72 (30.8%) as Grade B (moderate progression). The detailed clinical periodontal parameters at baseline, including mean CAL and PD, are provided in the **Table 1**.

**Table 1:** Baseline characteristics of the study cohort (N = 234).

Characteristic	Value
Demographics	
Age, years (mean ± SD)	68.4 ± 6.1
Sex, n (%)	
Male	130 (55.6)
Female	104 (44.4)
Diabetes-related parameters	
Diabetes Duration, years (mean ± SD)	8.5 ± 2.3
HbA1c, % (mean ± SD)	8.41 ± 0.52
Glycemic control category, n (%) [2]	
Moderate Control (HbA1c <7.5%)	56 (23.9)
Inadequate Control (HbA1c 7.5%–9.0%)	178 (76.1)
Periodontal clinical parameters (Mean ± SD)	
Probing Depth (PD), mm	6.2 ± 0.8
Clinical Attachment Loss (CAL), mm	5.1 ± 1.3
Bleeding on Probing (BOP), % of sites	58.7 ± 12.4
Periodontitis classification, n (%) [3]	
Stage	
Stage III	151 (64.5)
Stage IV	83 (35.5)
Grade	
Grade B	72 (30.8)
Grade C	162 (69.2)

### Gingival bleeding index and periodontitis grading before and after treatment

**Figure 1:**
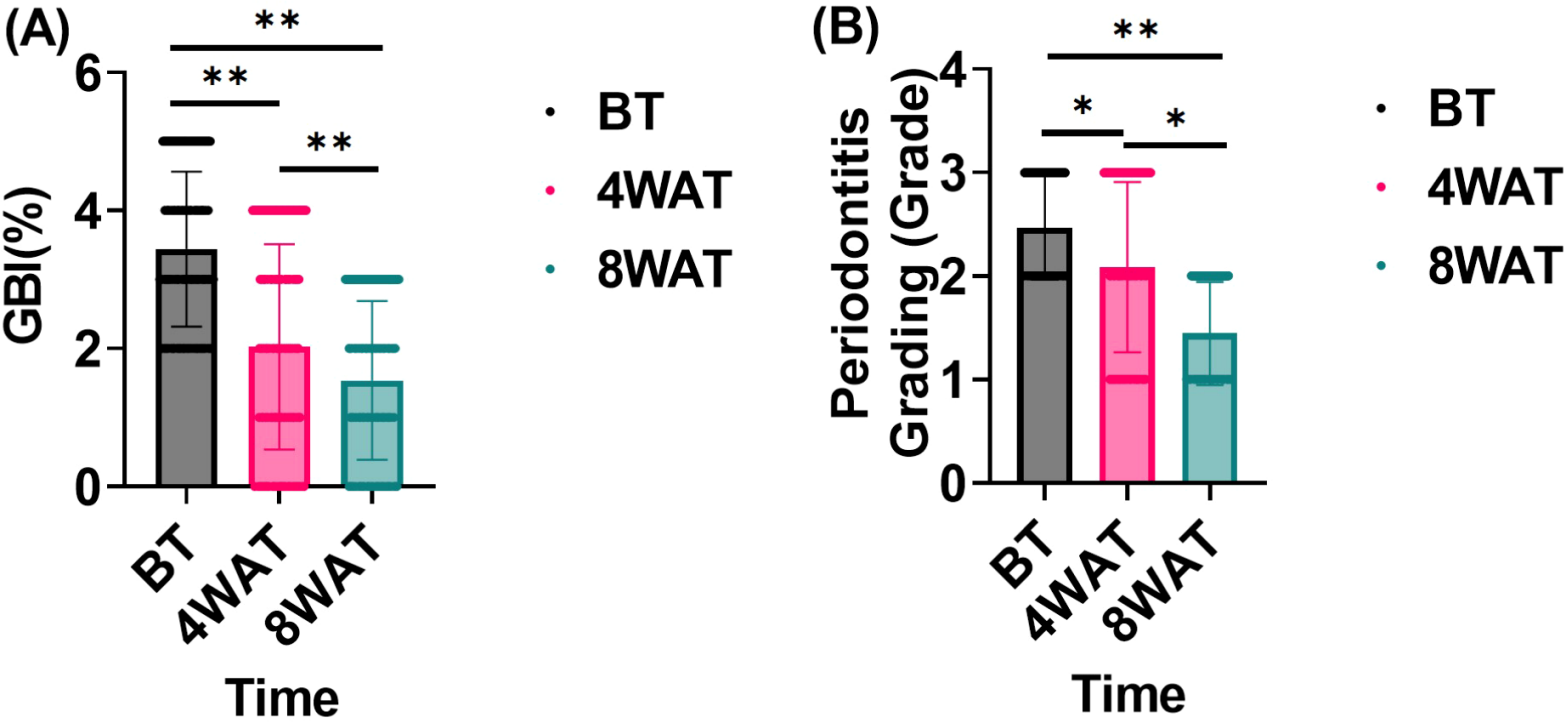
Gingival bleeding index and periodontitis grading before and after treatment. BT is Before Treatment,4WAT is 4 Weeks After Treatment,8WAT is 8 Weeks After Treatment. *P<0.05, **P<0.01. **(A)** GBI levels at different time points. Black dots represent BT, pink dots represent 4 Weeks After Treatment (4WAT), and blue dots represent 8WAT. **(B)** Periodontitis Grading at different time points. Black dots represent BT, pink dots represent 4WAT, and blue dots represent 8WAT.

In this study, we evaluated the effect of the treatment program on the improvement of periodontal health by comparing the changes in gingival bleeding index (GBI) and periodontitis grading before and after treatment. As can be seen in **Figure 1**, Prior to treatment, the subjects exhibited high GBI and periodontitis grading, indicative of poor periodontal health. After 4 weeks of treatment, both GBI and periodontitis grading significantly decreased, demonstrating a notable improvement in periodontal health. Further improvement was observed by 8 weeks post-treatment, with continued reductions in both GBI and periodontitis grading. These results highlight the efficacy of the treatment in improving gingival bleeding and reducing periodontitis grading over time.

### FBG and HbA1c levels before and after treatment

**Figure 2:**
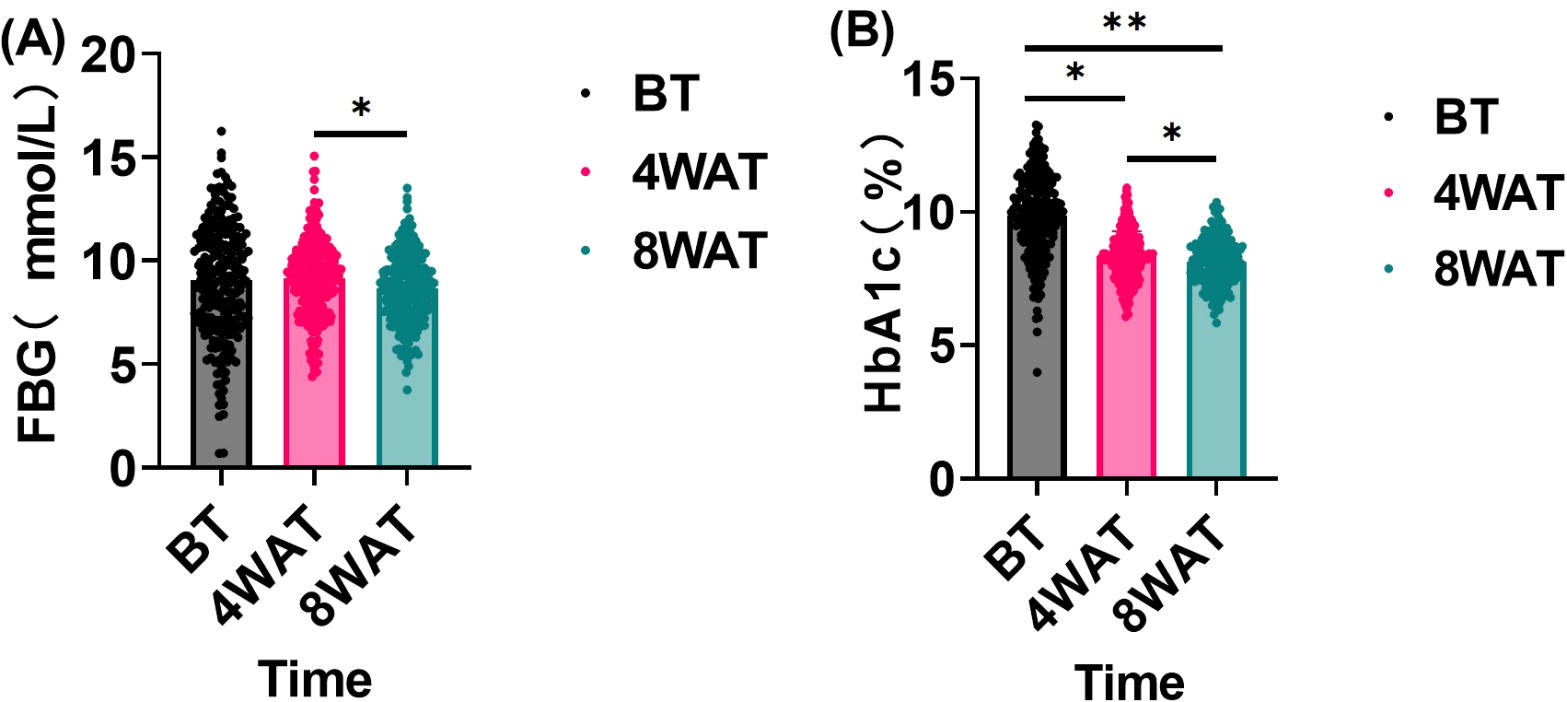
FBG and HbA1c levels before and after treatment(*n* = 234, `x ± s). BT is Before Treatment, 4WAT is 4 Weeks After Treatment, 8WAT is 8 Weeks After Treatment. *P<0.05, **P<0.01. **(A)** FBG levels at different time points. Black dots represent BT, pink dots represent 4WAT, and blue dots represent 8WAT. **(B)** HbA1c levels at different time points. Black dots represent BT, pink dots represent 4WAT, and blue dots represent 8WAT.

This study evaluated the effect of the treatment regimen on FBG and HbA1c levels in diabetic patients. As shown in **Figure 2**, These findings underscore the time-dependent efficacy of the treatment in enhancing glycemic control. The initial lack of significant change in FBG, despite a notable reduction in HbA1c at 4 weeks, suggests that the treatment’s impact on fasting blood glucose may require a longer duration to manifest fully. By 8 weeks, the significant decrease in both FBG and HbA1c indicates that the treatment not only sustained the initial improvement in HbA1c but also extended its beneficial effects to FBG, leading to a more comprehensive improvement in glycemic control. This progressive enhancement over time highlights the importance of continued treatment for optimal outcomes in managing glycemic parameters.

### Serum leptin, adiponectin and resistin levels before and after treatment

**Figure 3:**
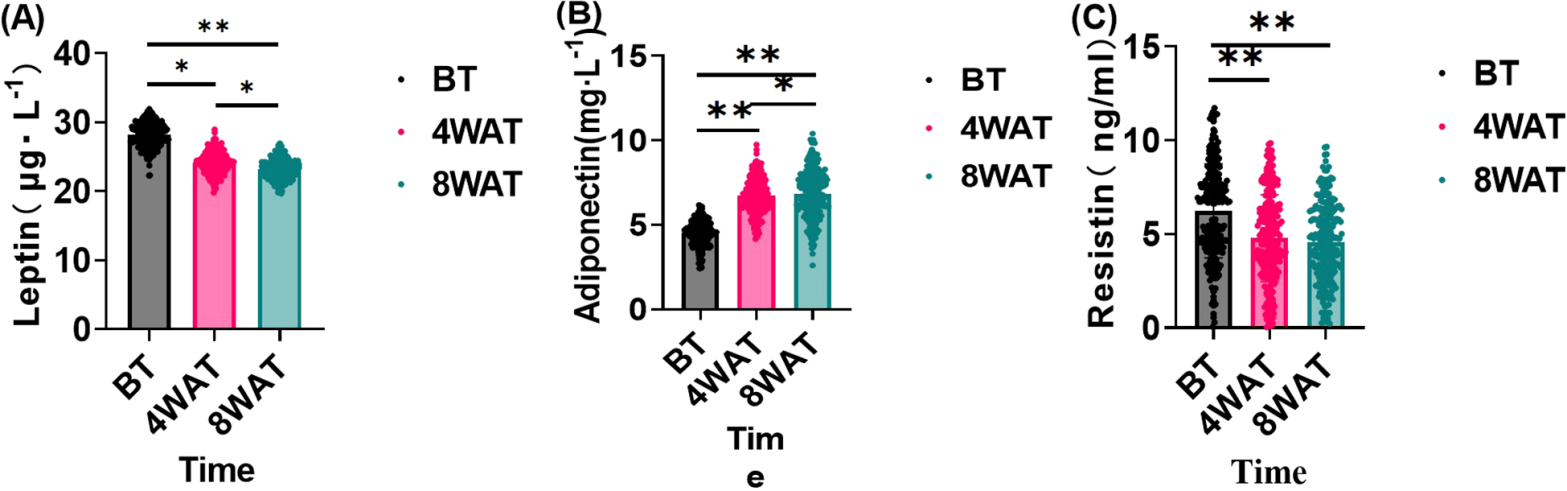
Changes in serum leptin, lipocalin and resistin before and after treatment. BT is Before Treatment, 4WAT is 4 Weeks After Treatment, 8WAT is 8 Weeks After Treatment. *P<0.05, **P<0.01. **(A)** Leptin levels at different time points. Black dots represent BT, pink dots represent 4WAT, and blue dots represent 8WAT. **(B)** Adiponectin levels at different time points. Black dots represent BT, pink dots represent 4WAT, and blue dots represent 8WAT. **(C)** Resistin levels at different time points. Black dots represent BT, pink dots represent 4WAT, and blue dots represent 8WAT.

This study further investigated the effects of the treatment regimen on leptin, adiponectin, and resistin levels in diabetic patients. As shown in **Figure 3**, The sustained and progressive changes in leptin, adiponectin, and resistin levels over the 8-week treatment period highlight the long-term efficacy of the treatment in modulating inflammatory and metabolic profiles. The initial significant reductions in leptin and resistin, coupled with the increase in adiponectin after 4 weeks, suggest a rapid onset of the treatment’s anti-inflammatory and metabolic regulatory effects. The further improvements observed at 8 weeks indicate that these effects are not only maintained but also enhanced with continued treatment. These results imply that the treatment has a cumulative impact on reducing inflammation and improving metabolic health, which is crucial for addressing underlying inflammatory and metabolic disorders.

### TNF-α and free fatty acid levels before and after treatment

**Figure 4:**
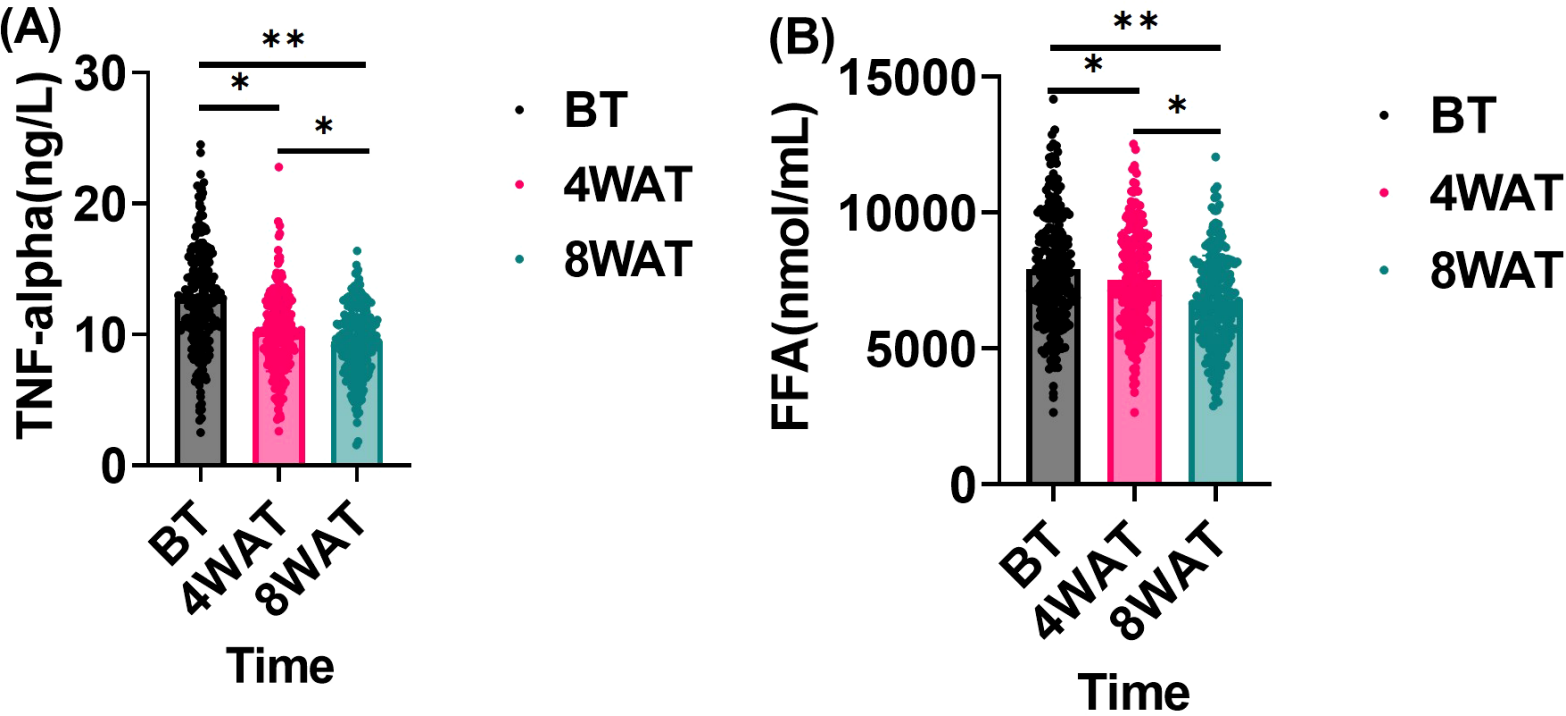
Changes in TNF-α and FFA before and after treatment. BT is Before Treatment, 4WAT is 4 Weeks After Treatment, 8WAT is 8 Weeks After Treatment. *P<0.05, **P<0.01. **(A)** TNF-α levels at different time points. Black dots represent BT, pink dots represent 4WAT, and blue dots represent 8WAT. **(B)** FFA levels at different time points. Black dots represent BT, pink dots represent 4WAT, and blue dots represent 8WAT.

This study investigated the effect of treatment regimen on TNF-α and FFA levels in diabetic patients. These indicators, which are closely related to inflammatory response and metabolic state, may impact periodontal health. As shown in **Figure 4**, At baseline, patients exhibited elevated levels of TNF-α and FFA, indicative of significant inflammatory responses and metabolic disorders. After 4 weeks of treatment, both TNF-α and FFA levels significantly decreased, demonstrating the treatment’s efficacy in modulating these markers in the short term. By 8 weeks, further reductions were observed, highlighting sustained and progressive improvements in inflammatory and metabolic status throughout the treatment period.

### Changes in inflammatory and metabolic indices within gingival crevicular fluid

Analysis of GCF provided insights into the local periodontal changes paralleling systemic effects. As detailed in **Table 2**, GCF levels of TNF-α, leptin, and resistin exhibited a significant and progressive decrease from baseline to 4 and 8 weeks post-treatment (all P < 0.01). Conversely, adiponectin levels in GCF showed a significant and sustained increase following therapy (P < 0.01). These local changes mirrored the systemic trends observed in serum, indicating that periodontal therapy effectively modulated the inflammatory and metabolic profile directly at the disease site. Furthermore, the reduction in GCF TNF-α at 8 weeks was strongly correlated with the concomitant reduction in serum TNF-α (ρ = 0.512, P < 0.01), reinforcing the link between local periodontal inflammation and systemic inflammatory burden.

**Table 2:** Dynamics of inflammatory and metabolic mediators in gingival crevicular fluid (GCF) following periodontal therapy.

Analyte (pg/μL)	Before treatment	4 2eeks after treatment	8 weeks after treatment
TNF-α (Mean ± SD)	18.5 ± 4.2	12.1 ± 3.0*	9.8 ± 2.6**
Leptin (Mean ± SD)	6.3 ± 1.5	4.8 ± 1.3*	3.9 ± 1.1**
Adiponectin (Mean ± SD)	1.05 ± 0.31	1.52 ± 0.40*	1.82 ± 0.45**
Resistin (Mean ± SD)	12.7 ± 3.1	9.2 ± 2.4*	7.4 ± 2.0**

*P < 0.05 vs. Baseline; **P < 0.01 vs. Baseline.

### Results of correlation analysis of indicators

**Figure 5:**
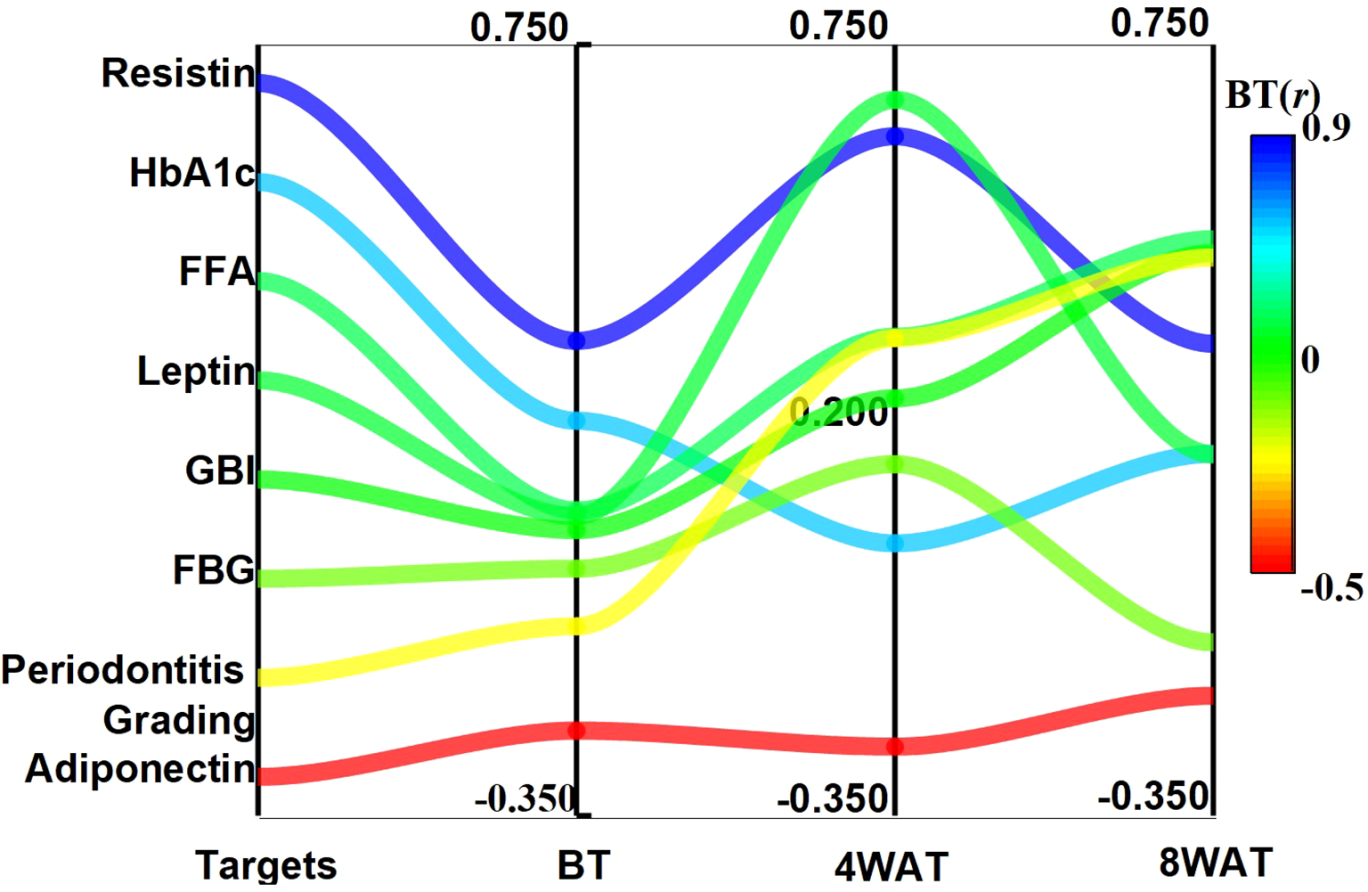
Trends of correlation coefficients between TNF-α and glycolipid metabolism and clinical indicators at different time points. BT is Before Treatment, 4WAT is 4 Weeks After Treatment, 8WAT is 8 Weeks After Treatment.

The temporal dynamics of TNF-α correlations with glycolipid metabolic and periodontal indices are summarized in **Table 3**. At baseline, TNF-α exhibited moderate positive associations with resistin (ρ = 0.327, P < 0.01) and HbA1c (ρ = 0.213, P < 0.01), while showing an inverse relationship with adiponectin (ρ = -0.229, P < 0.01). Following 4 weeks of therapy, TNF-α correlations intensified markedly with leptin (ρ = 0.671, P < 0.01), resistin (ρ = 0.619, P < 0.01), and periodontal indices (gingival bleeding index: ρ = 0.245; periodontitis grading: ρ = 0.331; P < 0.01). By 8 weeks, while associations with leptin (ρ = 0.165, P < 0.05) and adiponectin (ρ = -0.179, P < 0.01) attenuated, correlations with free fatty acids (FFA: ρ = 0.472) and periodontal severity (ρ = 0.446) became predominant (P < 0.01). Notably, resistin maintained the strongest persistent linkage to TNF-α across all phases (ρ > 0.323, P < 0.01), suggesting its potential role as a coregulated inflammatory mediator, as detailed in **Figure 5**.

**Table 3:** Spearman’s correlation coefficients (ρ) between TNF-**α** and glycolipid metabolic/periodontal/GCF indices across treatment phases.

Variable	TNF-α (ρ) before treatment	TNF-α (ρ) 4 weeks after treatment	TNF-α (ρ) 8 weeks after treatment
Serum & metabolic indices			
Resistin	0.327**	0.619**	0.323**
HbA1c	0.213**	0.038	0.166*
FFA	0.085	0.333**	0.472**
Leptin	0.077	0.671**	0.165*
Adiponectin	-0.229**	-0.252**	-0.179**
Clinical periodontal indices			
Gingival Bleeding Index (GBI)	0.057	0.245**	0.452**
FBG	0.002	0.151*	-0.103*
Periodontitis Grading	-0.080	0.331**	0.446**
GCF indices (TNF-α vs. GCF Analyte)			
GCF Leptin	0.463**	0.552**	0.581**
GCF Adiponectin	-0.401**	-0.455**	-0.498**
GCF Resistin	0.535**	0.648**	0.702**

*P < 0.05, **P < 0.01 (two-tailed, Benjamini-Hochberg corrected). GCF, Gingival Crevicular Fluid.

The correlation analysis was extended to the local periodontal microenvironment by examining the relationships between TNF-α and adipokines within the GCF (**Table 3**). Strong and persistent correlations were observed throughout the study period. At baseline, GCF TNF-α was already positively correlated with GCF leptin (ρ = 0.463) and GCF resistin (ρ = 0.535), and negatively correlated with GCF adiponectin (ρ = -0.401) (all P < 0.01). These associations intensified at the 4-week follow-up and remained the strongest of all measured correlations at the 8-week time point (GCF leptin: ρ = 0.581; GCF resistin: ρ = 0.702; GCF adiponectin: ρ = -0.498; all P < 0.01). The stability and magnitude of these relationships within the GCF highlight a tightly coupled network of inflammation and metabolism operating directly within the periodontal pocket.

## Discussion

An epidemiological survey indicates that from 2011 to 2020, the prevalence of dentate periodontitis among adults was approximately 62%, with severe periodontitis affecting 23.6% (35). In this study, through cross-sectional cohort research, we observed changes in periodontal health and metabolic indicators of patients with diabetes-associated periodontitis after systematic periodontal treatment. After 4-week and 8-week basic periodontal treatment, patients showed significant decreases in GBI and periodontitis grading. This demonstrates the treatment plan’s remarkable effectiveness in improving periodontal health. Our finding aligns with Menezes’s previous research (36), which suggests that systematic periodontitis treatment can effectively control periodontal inflammation and improve periodontal tissue health. Moreover, the treatment led to significant changes in metabolic indicators: decreased serum leptin levels, increased serum adiponectin levels, and decreased resistin levels (P < 0.05). These improvements persisted through the 8-week post-treatment period. This indicates that periodontal treatment enhances periodontal health and positively affects patients’ metabolic state. The research results suggest that periodontitis patients experience endoplasmic reticulum stress in their visceral fat cells, leading to hypoadiponectinemia (37). Adiponectin is an adipocytokine with anti-inflammatory and insulin-sensitizing effects. Higher adiponectin levels may improve insulin resistance and disorders of glucose and lipid metabolism (38). In patients with type 2 diabetes, the local inflammation of periodontitis leads to enhanced systemic inflammation, decreased adiponectin levels, and lipid metabolism disorders, creating an imbalance between pro-inflammatory and anti-inflammatory effects (39). This decrease in adiponectin levels in patients with diabetes and periodontitis strengthens the connection between these two conditions (40). Leptin, a peptide hormone, regulates energy balance, immune-inflammatory responses, and bone metabolism. Periodontitis progressively weakens tooth-supporting structures, ultimately causing tooth loss (41). Research with ob/ob mice has shown that leptin-deficient obesity increases the risk of periodontitis (42). Elevated levels of leptin and resistin may decrease inflammatory response and metabolic disorders, indicating how periodontal treatment helps regulate systemic metabolism. Consequently, periodontal therapy has been shown to enhance periodontal metabolism and reduce systemic inflammation and metabolic disorders in diabetic patients with periodontal disease.

Periodontal diseases arise from immune responses that trigger gingivitis, periodontitis, and their systemic effects (43). TNF-α, a key inflammatory cytokine, is strongly linked to insulin resistance, glucose metabolism disorders, and abnormal lipid metabolism. In our study, TNF-α levels decreased significantly after periodontal treatment (P < 0.05), suggesting that the treatment was associated with a reduction in inflammation. Both adipocytokines and oxidative stress play roles in periodontitis development (44, 45). The observation that demographic factors exclusively predict baseline TNF-α levels is noteworthy and can be contextualized within the framework of social determinants of health. These demographic variables often serve as proxies for cumulative life-course exposures, which shape an individual’s foundational inflammatory state. For instance, factors such as race, ethnicity, and socioeconomic environment are linked to disparities in baseline inflammatory profiles (46, 47). Research indicates that community-level disadvantages can drive increases in systemic inflammation over time (47). Furthermore, the influence of socioeconomic status on periodontal health may be mediated more by income and health behaviors than directly through inflammatory pathways (48). Therefore, it is plausible that chronic exposure to varying social and environmental stressors sets a distinct immunological tone, reflected in our baseline TNF-α measurements, before the acute effects of the intervention homogenize the inflammatory response.

The analysis of gingival crevicular fluid offers a unique window into the local periodontal biochemical milieu. Our findings demonstrate that non-surgical periodontal therapy induces a profound and progressive shift in the GCF profile, characterized by a significant reduction in pro-inflammatory (TNF-α) and metabolism-disrupting mediators (leptin, resistin), alongside an increase in the insulin-sensitizing adipokine adiponectin. These local changes, which temporally paralleled the systemic improvements in serum biomarkers, strongly suggest that the periodontal pocket is an active site of metabolic crosstalk. The significant correlation between the reduction of TNF-α in GCF and serum further solidifies the pathophysiological link between local periodontal inflammation and the systemic inflammatory state (49). This local modulation likely constitutes a pivotal initial step in the mechanistic pathway through which periodontal therapy confers its salutary systemic effects on glycolipid metabolism in diabetic patients.

Correlation analysis revealed that the relationships between TNF-α and indicators of glucose and lipid metabolism and related clinical indicators changed significantly during the treatment process. Four weeks after treatment, the correlations between TNF-α and leptin, adiponectin, resistin, FFA, the gingival bleeding index, and periodontitis grade were significantly greater (P < 0.05). These findings are consistent with TNF-α being involved in fat metabolism in the early treatment stages, likely because of its dual role in inflammation and metabolic regulation. Specifically, TNF-α is known to participate in inflammatory responses and influence metabolism by controlling adipocytokine expression. By eight weeks post-treatment, while some TNF-α correlations had weakened, their relationships with free fatty acids, the gingival bleeding index, and periodontitis grading strengthened. Correlation analysis revealed that the strengths of the associations between TNF-α and indicators of glucose/lipid metabolism and periodontal health changed during the treatment process. These observed shifts in correlation coefficients over time describe a pattern of changing associations, which may reflect the complex interplay between systemic inflammation and metabolism following periodontal intervention. However, as these are population-level correlations, they cannot be used to infer individual causal pathways. Notably, a significant correlation between TNF-α and resistin was observed, which persisted throughout the study period. This finding aligns with previous research by Zhang et al. (50). Resistin, an adipocyte-secreted hormone, is implicated in insulin resistance and inflammatory processes (51). The consistent association observed in our study invites the hypothesis that resistin and TNF-α may be interact within the shared inflammatory pathway of periodontitis and diabetes. This potential interaction warrants further investigation in future mechanistic studies designed to test causal relationships. Furthermore, the correlation analysis within GCF revealed a tightly interconnected network at the local disease site. The strong positive correlations of GCF TNF-α with GCF leptin and resistin, and its negative correlation with adiponectin, which were more pronounced than the systemic correlations, suggest a potent, localized interplay between inflammation and metabolism in the periodontium of diabetic patients. This reinforces the concept of the periodontal pocket as a significant site of pathophysiological crosstalk (52).

The results of this study indicate that periodontal treatment can significantly improve the periodontal health of diabetic patients and positively impact glucose and lipid metabolism by reducing TNF-α levels. This finding is closely related to the body’s inflammatory response, the regulatory role of adipocytokines, and the systemic metabolic state. For example, carriers of the TNF-α -308 G > A allele are more susceptible to apical periodontitis (53), leading to persistent periodontitis and maintaining the body in a state of chronic inflammation. The overexpression of inflammatory cytokines such as TNF-α can trigger a systemic inflammatory response, further exacerbating insulin resistance and metabolic disorders (54). Periodontal treatment may contribute to the reduction of this systemic inflammatory response and to improvements in glucose and lipid metabolism by removing periodontal inflammatory foci and decreasing TNF-α levels. Periodontal treatment also affects the serum levels of leptin, adiponectin, and resistin. These adipocytokines play crucial roles in insulin sensitivity and lipid metabolism. The increased adiponectin and decreased leptin and resistin levels may benefit glucose and lipid metabolism by enhancing insulin signaling and lipid metabolism pathways. Moreover, periodontal health is closely linked to the systemic metabolic state. Periodontitis influences metabolic processes through the inflammatory response and immune regulation. When periodontal treatment improves oral health, it may disrupt this harmful interaction, potentially promoting better metabolic function.

The selection of 4-week and 8-week post-treatment timepoints in our study was guided by a strategic consideration of inflammatory marker kinetics and established precedents in periodontal research involving patients with type 2 diabetes. We acknowledge that these intervals may not capture the most immediate transient peaks or very long-term effects; however, they are strategically positioned to assess the establishment of a new systemic inflammatory steady state. Following interventions such as non-surgical periodontal therapy, the initial response involves rapid fluctuations in upstream cytokines like TNF-α, which have a very short half-life (55). In contrast, more sustained systemic repercussions, reflected by downstream markers such as C-reactive protein (CRP), require several weeks to stabilize at a new, lower baseline, which genuinely signifies a shift in the patient’s inflammatory status (56). This intermediate observation window is consistent with high-quality evidence in the field. For instance, a randomized controlled trial by Yarahmadi et al. (2024) successfully demonstrated significant reductions in TNF-α and IL-6 at 8 weeks in diabetic patients with periodontal disease (57). Similarly, studies on other chronic inflammatory conditions, such as rheumatoid arthritis, commonly employ intervals of 4 to 12 weeks to assess the efficacy of anti-inflammatory interventions (58). Furthermore, while significant improvements in long-term glycemic control as measured by HbA1c may take several months to manifest (58), the early modulation of inflammation observed at 4 and 8 weeks serves as a crucial prognostic indicator for these ultimate metabolic benefits. Therefore, our chosen timepoints provide a scientifically grounded framework for evaluating the substantive impact of periodontal therapy on systemic inflammation.

This study has several limitations that should be considered when interpreting the results. The most significant limitation is the absence of a non-treatment or placebo control group. This design precludes us from definitively ruling out the possibility that the observed improvements reflect regression to the mean, natural fluctuation of the disease, or the effects of unmeasured confounders. However, several aspects of our data suggest that the changes are likely attributable to the treatment effect. First, the improvements were progressive and sustained over the 8-week observation period across a wide array of distinct biomarkers (from systemic cytokines to local GCF mediators) and clinical indices, a pattern less consistent with random fluctuation. Second, the observed temporal dynamics, particularly the intensification and subsequent evolution of correlations between TNF-α and metabolic markers, align with a biologically plausible sequence of events following the reduction of a key inflammatory driver (18–20). Finally, the magnitude and consistency of the metabolic improvements are in line with previous systematic reviews and meta-analyses of RCTs that have demonstrated the beneficial effect of periodontal therapy on glycemic control in diabetic patients (59, 60). Nonetheless, future randomized controlled trials with longer follow-up are warranted to conclusively confirm our findings. The 8-week follow-up period is relatively short, and the long-term sustainability of the observed metabolic and inflammatory improvements remains to be determined. Furthermore, potential confounders such as diet and physical activity were not systematically controlled or documented, and the influence of adjunctive tetracycline treatment cannot be excluded. Additionally, our study focused specifically on TNF-α as a primary mediator based on its well-established central role in the pathogenesis of both periodontitis and insulin resistance (61–66); however, we did not assess other pro-inflammatory cytokines (e.g., IL-1β, IL-6), which also participate in these processes. This focus, while providing depth to our understanding of TNF-α, means that the contributions of other inflammatory pathways remain unexplored in this cohort. Although the sample size was adequate, patient heterogeneity may exist. Finally, the specific mechanisms of action of TNF-α were not explored. Future studies with longer follow-up, randomized controlled designs, detailed monitoring of lifestyle factors, broader cytokine profiling, and integrated mechanistic investigations are warranted to validate and extend our findings.

## Conclusion

This study demonstrated that systematic periodontal treatment in diabetic patients significantly improves their periodontal health, blood glucose control, and glucose and lipid metabolism by reducing TNF-α levels. These findings provide an important foundation for treating patients with diabetes-complicated periodontitis. Future research should explore TNF-α’s mechanism in these patients to optimize treatment strategies and improve patient outcomes.

## Data Availability

The raw data supporting the conclusions of this article will be made available by the authors, without undue reservation.

## References

[1] Lee H Joo JY Song JM Kim HJ Kim YH Park HR. Immunological link between periodontitis and type 2 diabetes deciphered by single-cell RNA analysis. Clin Transl Med. (2023) 13:e1503. doi: 10.1002/ctm2.1503, PMID: 38082425 PMC10713875

[2] Li S Li H Kong H Wu SY Cheng CK Xu J. Endogenous and microbial biomarkers for periodontitis and type 2 diabetes mellitus. Front Endocrinol (Lausanne). (2023) 14:1292596. doi: 10.3389/fendo.2023.1292596, PMID: 38149100 PMC10750125

[3] Chung YL Lee JJ Chien HH Chang MC Jeng JH. Interplay between diabetes mellitus and periodontal/pulpal-periapical diseases. J Dent Sci. (2024) 19:1338–47. doi: 10.1016/j.jds.2024.03.021, PMID: 39035271 PMC11259663

[4] Aizenbud I Wilensky A Almoznino G. Periodontal disease and its association with metabolic syndrome-A comprehensive review. Int J Mol Sci. (2023) 24:13011. doi: 10.3390/ijms241613011, PMID: 37629193 PMC10455993

[5] Deng Y Xiao J Ma L Wang C Wang X Huang X. Mitochondrial dysfunction in periodontitis and associated systemic diseases: implications for pathomechanisms and therapeutic strategies. Int J Mol Sci. (2024) 25:1024. doi: 10.3390/ijms25021024, PMID: 38256098 PMC10816612

[6] Herrera D Sanz M Shapira L Brotons C Chapple I Frese T. Periodontal diseases and cardiovascular diseases, diabetes, and respiratory diseases: Summary of the consensus report by the European Federation of Periodontology and WONCA Europe. Eur J Gen Pract. (2024) 30:2320120. doi: 10.1080/13814788.2024.2320120, PMID: 38511739 PMC10962307

[7] Zhao X Yang Y Yu J Ding R Pei D Zhang Y. Injectable hydrogels with high drug loading through B-N coordination and ROS-triggered drug release for efficient treatment of chronic periodontitis in diabetic rats. Biomaterials. (2022) 282:121387. doi: 10.1016/j.biomaterials.2022.121387, PMID: 35093823

[8] Villoria G Fischer RG Tinoco E Meyle J Loos BG. Periodontal disease: A systemic condition. Periodontol. (2000) 2024. 96:7–19. doi: 10.1111/prd.12616, PMID: 39494478 PMC11579822

[9] Park MS Jeon J Song TJ Kim J. Association of periodontitis with microvascular complications of diabetes mellitus: A nationwide cohort study. J Diabetes Complications. (2022) 36:108107. doi: 10.1016/j.jdiacomp.2021.108107, PMID: 35063344

[10] Costa R Ríos-Carrasco B Monteiro L López-Jarana P Carneiro F Relvas M. Association between type 1 diabetes mellitus and periodontal diseases. J Clin Med. (2023) 12:1147. doi: 10.3390/jcm12031147, PMID: 36769794 PMC9917782

[11] Pinto KP Serrão G Alves Ferreira CM Sassone LM Fidalgo T Silva E. Association between apical periodontitis and chronic diseases: an umbrella review. Iran Endod J. (2023) 18:134–44. doi: 10.22037/iej.v18i3.42560, PMID: 37431524 PMC10329764

[12] Qin H Li G Xu X Zhang C Zhong W Xu S. The role of oral microbiome in periodontitis under diabetes mellitus. J Oral Microbiol. (2022) 14:2078031. doi: 10.1080/20002297.2022.2078031, PMID: 35694215 PMC9176325

[13] Tang B Yan C Shen X Li Y. The bidirectional biological interplay between microbiome and viruses in periodontitis and type-2 diabetes mellitus. Front Immunol. (2022) 13:885029. doi: 10.3389/fimmu.2022.885029, PMID: 36131931 PMC9483123

[14] Păunică I Giurgiu M Dumitriu AS Păunică S Pantea Stoian AM Martu MA. The bidirectional relationship between periodontal disease and diabetes mellitus-A review. Diagnostics (Basel). (2023) 13:681. doi: 10.3390/diagnostics13040681, PMID: 36832168 PMC9954907

[15] Li LL Xie XT Wu Y Yan FH. Advances in research on the mechanism of association between periodontitis and diabetes mellitus. Sichuan Da Xue Bao Yi Xue Ban. (2023) 54:71–6. doi: 10.12182/20230160203, PMID: 36647646 PMC10409046

[16] Yang Y Sun X Yang Y Qie Y. Insight of the interrelationship and association mechanism between periodontitis and diabetes mellitus. Regener Ther. (2024) 26:1159–67. doi: 10.1016/j.reth.2024.11.001, PMID: 39640921 PMC11617686

[17] Elnour M Mirghani HO. Periodontitis treatment (surgical and nonsurgical) effects on glycemic control: A review and meta-analysis. Ann Afr Med. (2023) 22:131–5. doi: 10.4103/aam.aam_53_22, PMID: 37026192 PMC10262851

[18] Prasetyo EP Sampoerno G Juniarti DE Cahyani F Saraswati W Kuntjoro M. Effect of lipopolysaccharide-induced apical periodontitis in diabetes mellitus rats on periapical inflammation. Eur J Dent. (2023) 17:1146–52. doi: 10.1055/s-0042-1758790, PMID: 36599453 PMC10756800

[19] Akash M Rehman K Liaqat A. Tumor necrosis factor-α: role in development of insulin resistance and pathogenesis of type 2 diabetes mellitus. J Cell Biochem. (2018) 119:105–10. doi: 10.1002/jcb.26174, PMID: 28569437

[20] Marigo L Cerreto R Giuliani M Somma F Lajolo C Cordaro M. Diabetes mellitus: biochemical, histological and microbiological aspects in periodontal disease. Eur Rev Med Pharmacol Sci. (2011) 15:751–8., PMID: 21780542

[21] Bakshi D Kaur G Singh D Sahota J Thakur A Grover S. Estimation of plasma levels of tumor necrosis factor-a, interleukin-4 and 6 in patients with chronic periodontitis and type II diabetes mellitus. J Contemp Dent Pract. (2018) 19:166–9. doi: 10.5005/jp-journals-10024-2231, PMID: 29422465

[22] Palafox-Sánchez CA Cruz A Salazar-Camarena DC Gascón LG Cintra LTA Muñoz-Valle JF. Evaluation of serum levels of cytokines in acute apical abscess: A longitudinal observational study. J Endod. (2023) 49:1090–8. doi: 10.1016/j.joen.2023.07.001, PMID: 37423583

[23] Chaudhry SZ Ghafoor S. Adipokines: Diagnostic and prognostic markers for oral diseases. J Pak Med Assoc. (2023) 73:858–62. doi: 10.47391/JPMA.4737, PMID: 37052000

[24] American Diabetes Association Professional Practice Committee. 2. Classification and diagnosis of diabetes: standards of medical care in diabetes—2022. Diabetes Care. (2022) 45:S17–38. doi: 10.2337/dc22-S002, PMID: 34964875

[25] American Diabetes Association Professional Practice Committee. 6. Glycemic targets: standards of medical care in diabetes—2022. Diabetes Care. (2022) 45:S83–96. doi: 10.2337/dc22-S006, PMID: 34964868

[26] Tonetti MS Greenwell H Kornman KS. Staging and grading of periodontitis: Framework and proposal of a new classification and case definition. J Periodontol. (2018) 89 Suppl 1:S159–72. doi: 10.1002/JPER.18-0006, PMID: 29926952

[27] Giannobile WV Burt BA Genco RJ eds. Design of randomized controlled trials. In: Clinical research in oral health. Hoboken, NJ: John Wiley & Sons (2010). 119–36.

[28] Muramatsu T Yokoyama Y Yatani H Kado T Koso K Hanioka T. Team medical approach for drug-induced gingival overgrowth. J Jpn Soc Periodontol. (2019) 61:37–43. doi: 10.1155/crid/8318894, PMID: 39968166 PMC11835479

[29] American Diabetes Association Professional Practice Committee. 10. Cardiovascular disease and risk management: Standards of Care in Diabetes—2024. Diabetes Care. (2024) 47:S179–218. doi: 10.2337/dc24-S010, PMID: 38078592 PMC10725811

[30] Wu SX. Sample size calculation basics for clinical research. Beijing: People’s Medical Publishing House (2008) p. 24–6.

[31] Narayanan S. Preanalytical variables in biological monitoring. Pure Appl Chem. (2000) 72:1487–510. doi: 10.1351/pac200072081487

[32] Griffiths GS. Formation, collection and significance of gingival crevice fluid. Periodontol. (2000) 2003:31:32–42. doi: 10.1034/j.1600-0757.2003.03103.x, PMID: 12656994

[33] Abrahamian L Pascual-LaRocca A Barallat L Valles C Herrera D Sanz M. Intra- and inter-examiner reliability in classifying periodontitis according to the 2018 classification of periodontal diseases. J Clin Periodontol. (2022) 49:457–64. doi: 10.1111/jcpe.13604, PMID: 35322458 PMC9545414

[34] Mühlemann HR Son S. Gingival sulcus bleeding–a leading symptom in initial gingivitis. Helv Odontol Acta. (1971) 15:107–13., PMID: 5315729

[35] Trindade D Carvalho R MaChado V Chambrone L Mendes JJ Botelho J. Prevalence of periodontitis in dentate people between 2011 and 2020: A systematic review and meta-analysis of epidemiological studies. J Clin Periodontol. (2023) 50:604–26. doi: 10.1111/jcpe.13769, PMID: 36631982

[36] Menezes CC Barbirato D Fogacci MF Marañón-Vásquez GA Carneiro JRI Maia LC. Systemic benefits of periodontal therapy in patients with obesity and periodontitis: a systematic review. Braz Oral Res. (2024) 38:e031. doi: 10.1590/1807-3107bor-2024.vol38.0031, PMID: 38597549 PMC11376685

[37] Wu Q Yan L Wu X Chen Y Ye L Lv Y. Experimental periodontitis induced hypoadiponectinemia by IRE1α-mediated endoplasmic reticulum stress in adipocytes. BMC Oral Health. (2023) 23:1032. doi: 10.1186/s12903-023-03758-6, PMID: 38129878 PMC10740306

[38] Sun Y Yin Y Yang S Ai D Qin H Xia X. Lipotoxicity: The missing link between diabetes and periodontitis. J Periodontal Res. (2024) 59:431–45. doi: 10.1111/jre.13242, PMID: 38419425

[39] Cheng R Xu X Yang S Mi Z Zhao Y Wang C. The effect of APN, hs-CRP and APN/hs-CRP in periodontitis with DAA. BMC Oral Health. (2023) 23:85. doi: 10.1186/s12903-023-02765-x, PMID: 36765308 PMC9921664

[40] Mahendra J Mahendra L Divya D Ilango P Devarajan N Thanigaimalai A. Association of Epstein-Barr virus, cytomegalovirus and lipocalin with periodontitis in type 2 diabetic subjects. Oral Dis. (2023) 29:1163–71. doi: 10.1111/odi.14091, PMID: 34850506

[41] Guo Z Peng Y Hu Q Liu N Liu Q. The relationship between leptin and periodontitis: a literature review. PeerJ. (2023) 11:e16633. doi: 10.7717/peerj.16633, PMID: 38111655 PMC10726740

[42] Li Z Zheng Z Pathak JL Li H Wu G Xu S. Leptin-deficient ob/ob mice exhibit periodontitis phenotype and altered oral microbiome. J Periodontal Res. (2023) 58:392–402. doi: 10.1111/jre.13099, PMID: 36710264

[43] Zhang M Liu Y Afzali H Graves DT. An update on periodontal inflammation and bone loss. Front Immunol. (2024) 15:1385436. doi: 10.3389/fimmu.2024.1385436, PMID: 38919613 PMC11196616

[44] Iniesta M Chamorro C Ambrosio N Marín MJ Sanz M Herrera D. Subgingival microbiome in periodontal health, gingivitis and different stages of periodontitis. J Clin Periodontol. (2023) 50:905–20. doi: 10.1111/jcpe.13793, PMID: 36792073

[45] Sabbagh S Adatorwovor R Kirakodu S Rojas-Ramirez MV Al-Sabbagh M Dawson D. Periodontal inflammatory and microbial profiles in healthy young African Americans and Caucasians. J Clin Periodontol. (2024) 51:905–14. doi: 10.1111/jcpe.13989, PMID: 38763508 PMC11182714

[46] GogniatMAKhanOARatangeeBBoltonCJZhangPLiuD. Cross-sectional and longitudinal associations of neighborhood disadvantage with fluid biomarkers of neuroinflammation and neurodegeneration. Neurology. (2025) 105:e214280. doi: 10.1212/WNL.0000000000213770, PMID: 40982775

[47] Chen Y Wang L Mo S Zhao D Fan Y. Mediators of the association between education and periodontitis: Mendelian randomization study. BMC Oral Health. (2025) 25:159. doi: 10.1186/s12903-025-06006-1, PMID: 40287678 PMC12034195

[48] Toy VE Ataoglu T Eltas A Otlu HG Karabulut AB. Obesity as a modifying factor of periodontal therapy outcomes: local and systemic adipocytokines and oxidative stress markers. Clin Oral Investig. (2023) 27:2763–73. doi: 10.1007/s00784-022-04854-7, PMID: 36604342

[49] Hajishengallis G Chavakis T. Local and systemic mechanisms linking periodontal disease and inflammatory comorbidities. Nat Rev Immunol. (2021) 21:426–40. doi: 10.1038/s41577-020-00488-6, PMID: 33510490 PMC7841384

[50] Zhang Y Jia R Zhang Y Sun X Mei Y Zou R. Effect of nonsurgical periodontal treatment on cytokines/adipocytokines levels among periodontitis patients with or without obesity: a systematic review and meta-analysis. BMC Oral Health. (2023) 23:717. doi: 10.1186/s12903-023-03383-3, PMID: 37798684 PMC10552206

[51] Kamil MA Peeran SW Basheer SN Elhassan A Alam MN Thiruneervannan M. Role of resistin in various diseases with special emphasis on periodontal and periapical inflammation - A review. J Pharm Bioallied Sci. (2023) 15:S31–5. doi: 10.4103/jpbs.jpbs_655_22, PMID: 37654317 PMC10466674

[52] Fischer RG Lira Junior R Retamal-Valdes B Figueiredo LC Malheiros Z Stewart B. Periodontal disease and its impact on general health in Latin America. Section V: Treatment of periodontitis. Braz Oral Res. (2020) 34:e026. doi: 10.1590/1807-3107bor-2020.vol34.0026, PMID: 32294679

[53] Jakovljevic A Jacimovic J Georgiou AC Nikolic N Aminoshariae A van der Waal SV. Single nucleotide polymorphisms as a predisposing factor for the development of apical periodontitis-An umbrella review. Int Endod J. (2022) 55:700–13. doi: 10.1111/iej.13756, PMID: 35476797

[54] Kandaswamy E Lee CT Gururaj SB Shivanaikar S Joshi VM. Association of adipokine levels with obesity in periodontal health and disease: A systematic review with meta-analysis and meta-regression. J Periodontal Res. (2024) 59:623–35. doi: 10.1111/jre.13263, PMID: 38594806

[55] Cavaillon JM Adib-Conquy M Fitting C Adrie C Monchi M. Cytokine cascade in sepsis. Scand J Infect Dis. (2003) 35:535–44. doi: 10.1080/00365540310015935, PMID: 14620132

[56] Aispure O Ivanov D Smirnov A Lazko F Ryabykh S Guz A. Dynamics of C-reactive protein level after orthopedic surgeries. Int J Surg. (2024) 11:7., PMID: 38046451

[57] Yarahmadi M Zeynali N Zadeh F Yousefimanesh HA Nejatian T Gravand E. The effects of synbiotic supplementation along with non-surgical periodontal therapy in improving metabolic status and inflammatory markers in type 2 diabetes mellitus patients with periodontal disease: A double-blind randomized clinical trial. J Educ Health Promot. (2024) 13:430. doi: 10.4103/jehp.jehp_1382_23, PMID: 39811858 PMC11731246

[58] D'Aiuto F Gkranias N Bhowruth D Horváth Á Szentpéteri A Hamar A. Systemic effects of periodontitis treatment in patients with type 2 diabetes: a 12 month randomised controlled clinical trial. Lancet Diabetes Endocrinol. (2018) 6:954–65. doi: 10.1016/S2213-8587(18)30038-X, PMID: 30472992

[59] Sanz M Ceriello A Buysschaert M Khan T Orlandi M Suvan J. Scientific evidence on the links between periodontal diseases and diabetes: Consensus report and guidelines of the joint workshop on periodontal diseases and diabetes by the International diabetes Federation and the European Federation of Periodontology. Diabetes Res Clin Pract. (2018) 137:231–41. doi: 10.1016/j.diabres.2017.12.001, PMID: 29208508

[60] Teshome A Yitayeh A. The effect of periodontal therapy on glycemic control and fasting plasma glucose level in type 2 diabetic patients: systematic review and meta-analysis. BMC Oral Health. (2016) 17:31. doi: 10.1186/s12903-016-0249-1, PMID: 27473177 PMC4967318

[61] Graves DT Cochran D. The contribution of interleukin-1 and tumor necrosis factor to periodontal tissue destruction. J Periodontol. (2003) 74:391–401. doi: 10.1902/jop.2003.74.3.391, PMID: 12710761

[62] Hotamisligil GS Shargill NS Spiegelman BM. Adipose expression of tumor necrosis factor-alpha: direct role in obesity-linked insulin resistance. Science. (1993) 259:87–91. doi: 10.1126/science.7678183, PMID: 7678183

[63] Krogh-Madsen R Plomgaard P Møller K Mittendorfer B Pedersen BK. Influence of TNF-alpha and IL-6 infusions on insulin sensitivity and expression of IL-18 in humans. Am J Physiol Endocrinol Metab. (2006) 291:E108–14. doi: 10.1152/ajpendo.00471.2005, PMID: 16464907

[64] Ding C Ji X Chen X Xu Y Zhong L. TNF-α gene promoter polymorphisms contribute to periodontitis susceptibility: evidence from 46 studies. J Clin Periodontol. (2014) 41:748–59. doi: 10.1111/jcpe.12279, PMID: 24905365

[65] Li Y Yang J Wu X Sun W. TNF-α polymorphisms might influence predisposition to periodontitis: A meta-analysis. Microb Pathog. (2020) 143:104113. doi: 10.1016/j.micpath.2020.104113, PMID: 32130979

[66] Czókolyová M Pusztai A Végh E Horváth Á Szentpéteri A Hamar A. Changes of metabolic biomarker levels upon one-year anti-TNF-α Therapy in rheumatoid arthritis and ankylosing spondylitis: associations with vascular pathophysiology. Biomolecules. (2021) 11:1535. doi: 10.3390/biom11101535, PMID: 34680168 PMC8533731

